# Beyond approval: a mechanism-based review of novel anti-tumour agents in cervical cancer and when to use them

**DOI:** 10.3389/fimmu.2026.1948148

**Published:** 2026-09-07

**Authors:** Chenguang Yang, Xuan Song, Hongmei Sun, Min Chen, Xialing Zhu, Xi Chen

**Affiliations:** 1 Department of Gynaecology and Obstetrics, Affiliated Xuancheng Hospital of Wannan Medical University, Xuancheng, Anhui, China; 2 Department of General Medicine, Affiliated Xuancheng Hospital of Wannan Medical University, Xuancheng, Anhui, China; 3 Department of Epidemiology and Statistics, School of Public Health, Medical College, Zhejiang University, Hangzhou, China

**Keywords:** antibody–drug conjugates, cervical cancer, immune checkpoint inhibitors, novel systemic therapy, predictive biomarkers, treatment sequencing

## Abstract

Cervical cancer remains a leading cause of cancer death in women, and outcomes in recurrent or metastatic disease remain poor despite chemoradiation and bevacizumab. The HPV-driven biology that defines the disease—viral E6/E7 oncoproteins, an inflamed microenvironment and exploitable surface antigens—has enabled a rapid expansion of novel systemic therapies, yet existing reviews tend to catalogue these agents by class or report individual trials in isolation, leaving their comparative efficacy, safety and clinical positioning unresolved. Here we organise emerging anti-tumour agents by mechanism of action and array them along a translational maturity gradient, from approved regimens through phase 1/2 candidates to preclinical assets, appraising efficacy and safety in parallel. We cover immune checkpoint inhibitors, bispecific antibodies, antibody–drug conjugates (ADCs), therapeutic HPV vaccines and adoptive cell therapy, and integrate China-developed agents—including the PD-1/CTLA-4 bispecific cadonilimab and the Nectin-4 conjugate 9MW2821—into the global landscape. Departing from a purely descriptive account, we compare class-specific toxicity profiles and predictive biomarkers side by side and, most importantly, translate the fragmented evidence into a resistance-directed treatment framework anchored on platinum-resistant disease. We further argue that, because access to newly approved agents varies widely between health systems, the line of therapy at which an agent is introduced—rather than its approval status alone—may influence survival, a decision-relevant question that current guidelines leave unanswered. This mechanism-based, decision-oriented synthesis is intended to guide rational agent selection, sequencing and combination strategy across diverse clinical settings.

## Highlights

- Checkpoint inhibitors, ADCs and bispecifics are compared on efficacy and safety.
- China-developed cadonilimab and Nectin-4 ADC 9MW2821 are integrated into practice.
- Class-specific resistance and toxicity profiles guide rational agent selection.
- A resistance-directed sequence anchors therapy in platinum-resistant disease.
- Line of therapy and global access shape survival beyond approval status.

## 1 Introduction

Cervical cancer remains a leading cause of cancer death amongst women, with an estimated 662,000 new cases and 349,000 deaths worldwide in 2022, the overwhelming majority concentrated in low- and middle-income countries (1). Nearly all cases are driven by persistent high-risk human papillomavirus (HPV) infection, in which the viral oncoproteins E6 and E7 disable the tumour-suppressor axis: E6 targets p53 for ubiquitin-mediated degradation (2), whilst E7 inactivates the retinoblastoma protein (3). This obligate viral dependency also renders the tumour immunologically distinct. Clinically, outcomes diverge sharply by stage: locally advanced disease is managed with curative intent, whereas recurrent or metastatic (R/M) disease carries a dismal prognosis (4), a divide that continues to shape both trial design and the elimination targets set by the World Health Organization (5).

For two decades, therapeutic progress was incremental. Concurrent cisplatin-based chemoradiation, established by a cluster of randomised trials in 1999, became the backbone for locally advanced disease (6, 7), yet a substantial fraction of patients relapse. In the R/M setting, the addition of bevacizumab to platinum–paclitaxel (GOG-240) extended median overall survival from 13.3 to 17.0 months, the first non-cytotoxic gain in this population, but at the cost of characteristic fistula formation and an efficacy ceiling that left most patients without durable control (8, 9). These limits defined a persistent unmet need and set the stage for mechanistically novel agents.

The same HPV-driven biology that causes cervical cancer also makes it an attractive target for immune and precision therapies. Genomic characterisation has revealed APOBEC-driven mutagenesis, frequent PD-L1 amplification and an inflamed, immunogenic microenvironment (10), whilst constitutively expressed E6/E7 antigens provide tumour-specific targets absent from normal tissue. This rationale has been validated in rapid succession. First-line pembrolizumab added to chemotherapy (KEYNOTE-826) improved both progression-free and overall survival, redefining the standard of care in R/M disease (11). The antibody–drug conjugate (ADC) tisotumab vedotin then confirmed the platform, reducing the risk of death by 30% in previously treated patients (innovaTV 301) (12). This result is underpinned by the broad expression of tissue factor and of Trop-2 and Nectin-4 across cervical tumours, which now defines an expanding target landscape (13).

Building on these advances, a diverse pipeline has emerged spanning immune checkpoint inhibitors, bispecific antibodies, ADCs, therapeutic HPV vaccines and adoptive cell therapy (**Table 1**). China-developed agents have moved to this frontier: cadonilimab, the first PD-1/CTLA-4 bispecific antibody to reach a positive first-line phase 3 trial (COMPASSION-16), exemplifies the shift (14). Yet the evidence remains fragmented across drug classes and stages of development, and the class-specific toxicity profiles—ranging from immune-related adverse events to ocular and haemorrhagic events—together with their predictive biomarkers, are rarely compared side by side.

**Table 1:** Emerging (phase 1/2) and preclinical agents, stratified by mechanism class.

Mechanism class	Agent	Molecular target	Development stage	Preliminary efficacy signal (verified)	Representative trial/NCT	Origin	Ref.
ICI	Nivolumab	PD-1	Phase 1/2	Cervical-cohort ORR 26.3% (5/19); mOS 21.9 mo	CheckMate 358/NCT02488759	—	(21)
ICI	Durvalumab + CCRT	PD-L1	Phase 3	Did not meet primary PFS endpoint (negative; instructive for design)	CALLA/NCT03830866	—	(24)
ICI	Ociperlimab + tislelizumab	TIGIT	Phase 2 (ongoing)	Proof-of-concept against adaptive resistance; results awaited	AdvanTIG-202/NCT04693234	CN	—
Bispecific	Balstilimab + zalifrelimab	PD-1 + CTLA-4	Phase 2	2L ORR 25.6% (PD-L1 + 32.8%/PD-L1− 9.1%; squamous 32.6%); DCR 52%	NCT03495882	—	(37, 38)
ADC	Trastuzumab deruxtecan	HER2 (topoisomerase-I payload)	Phase 2	Cervical-cohort ORR 50.0% (20/40); basket ORR 37.1%, central IHC3 + 61.3%	DESTINY-PanTumor02/NCT04482309	—	(41–43)
ADC	9MW2821	Nectin-4 (MMAE)	Phase 1/2	Preclinical activity ≥ enfortumab vedotin (CDX/PDX); now in phase 1/2	NCT05216965/NCT05773937	CN	(46, 47)
ADC	Sacituzumab govitecan	Trop-2 (topoisomerase-I payload)	Preclinical	Cervical-model activity rising with Trop-2 expression	—	—	(48)
Therapeutic vaccine	VGX-3100	HPV-16/18 E6/E7 (DNA)	Phase 2b	CIN2/3 histological regression 49.5% vs 30.6% placebo (per-protocol, p=0.034)	NCT01304524	—	(52)
Therapeutic vaccine	VB10.16	HPV-16 E6/E7 (APC-targeted DNA)	Phase 1/2a	High-grade CIN: HPV-16 clearance 47% (8/17); regression to CIN0/1 59% (10/17)	—	—	(53)
Therapeutic vaccine	GX-188E + pembrolizumab	HPV E6/E7 (DNA) + PD-1	Phase 2	Advanced disease ORR 42% (11/26; CR 15%, PR 27%)	NCT03444376	CN	(59)
Therapeutic vaccine	Axalimogene filolisbac	Listeria-vectored E7	Phase 2	Platinum-refractory 12-mo OS 38% (met benchmark); mOS 6.1 mo, mPFS 2.8 mo	GOG-0265/NCT01266460	—	(58)
Adoptive cell therapy	HPV-reactive TIL	HPV E6/E7 (polyclonal)	Phase 2	Cervical-cohort ORR 28% (5/18); durable complete responses ongoing at 53–67 mo	NCI Surgery Branch/NCT01585428	—	(60, 61)
Adoptive cell therapy	E7 TCR-T	HPV-16 E7 epitope	Phase 1	Objective responses in 6/12, including 4/8 refractory to prior anti–PD-1	NCT02858310	—	(62)
Adoptive cell therapy	CAR-T	Surface antigen	Preclinical	Limited by intracellular antigens/MHC presentation (no cervical clinical data)	—	—	—
Combination	Camrelizumab + famitinib	PD-1 + antiangiogenic TKI	Phase 2	R/M squamous ORR 39.4% (13/33); mPFS 10.3 mo; 12-mo OS 77.7%	NCT03827837	CN	(63)
Combination	Sintilimab + anlotinib	PD-1 + antiangiogenic TKI	Phase 2	PD-L1 + 2L ORR 54.8%; mPFS 9.4 mo; durable responses on extended follow-up (65)	ChiCTR1900023015	CN	(64, 65)
Combination	Serplulimab + nab-paclitaxel	PD-1 + chemotherapy	Phase 2	PD-L1+ pretreated ORR 57.1% (IRRC); mPFS 5.7 mo; mOS 15.5 mo	NCT04150575	CN	(66)
Combination	Sintilimab + nab-paclitaxel	PD-1 + chemotherapy	Phase 2	R/M ORR 44.4%; DCR 88.9%; mPFS 5.2 mo; mOS 13.1 mo	NCT04341883	CN	(67)

ICI, immune checkpoint inhibitor; ADC, antibody–drug conjugate; TKI, tyrosine kinase inhibitor; TIL, tumour-infiltrating lymphocyte; TCR-T, T-cell receptor–engineered T cell; CAR-T, chimeric antigen receptor T cell; CIN, cervical intraepithelial neoplasia; CDX/PDX, cell-line/patient-derived xenograft; IRRC, independent radiological review committee; DCR, disease control rate; IHC, immunohistochemistry; CR, complete response; PR, partial response. The sintilimab + anlotinib trial was registered in the Chinese Clinical Trial Registry (ChiCTR1900023015) rather than ClinicalTrials.gov.

**Figure 1:**
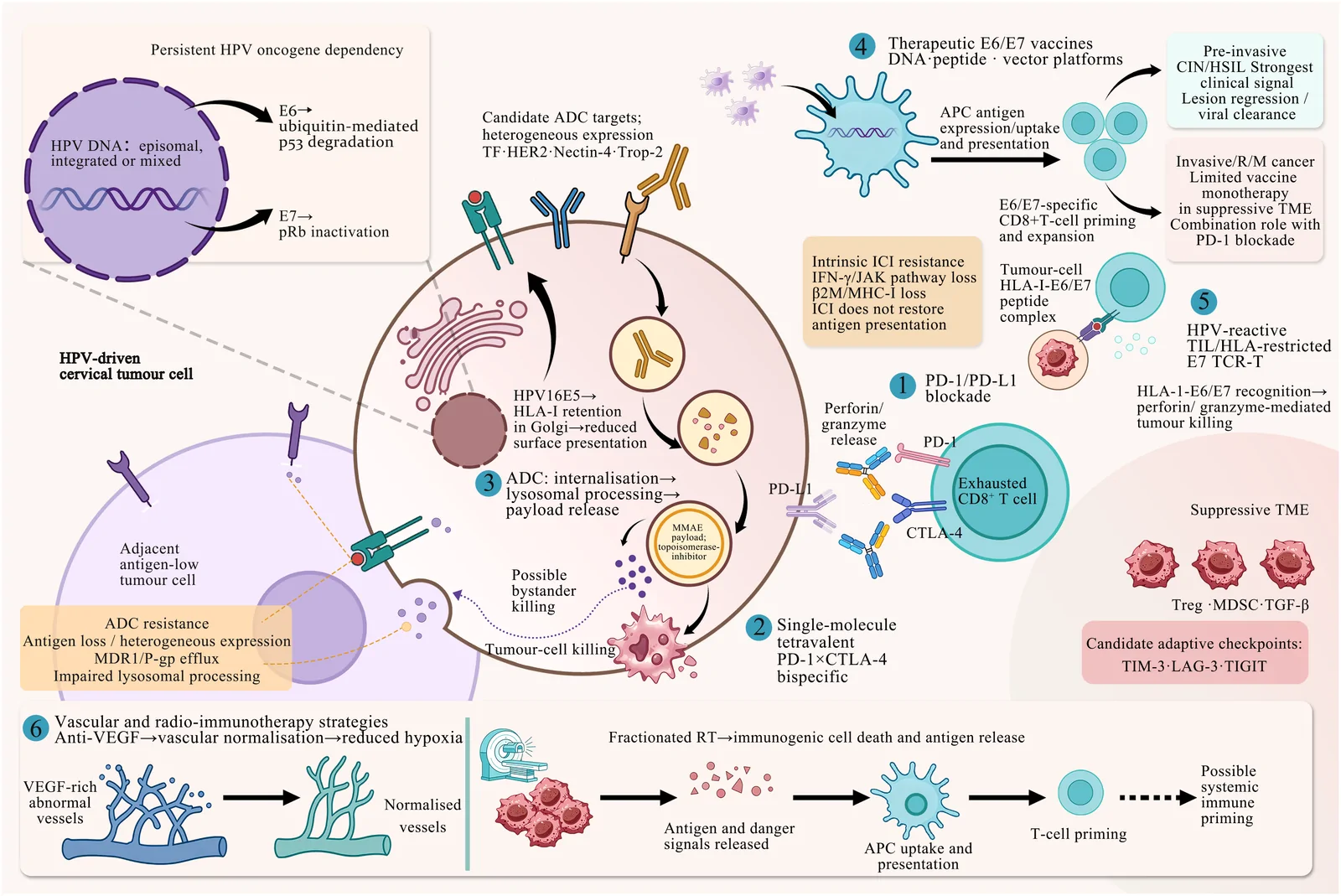
Six mechanistically distinct agent classes converge on HPV-driven cervical cancer biology. Persistent high-risk human papillomavirus (HPV) drives cervical carcinogenesis through E6-mediated degradation of p53 and E7-mediated inactivation of the retinoblastoma protein, whilst E5 retains HLA class I intracellularly to impair antigen presentation. Together these events generate an immunogenic yet functionally exhausted tumour microenvironment and a defined repertoire of targetable surface antigens (tissue factor, HER2, Nectin-4 and Trop-2). Numbered insets (①–⑥) map each of the six agent classes to its molecular site of action: ① immune checkpoint inhibitors, which restore exhausted CD8^+^ T-cell function by blocking the PD-1/PD-L1 axis; ② PD-1×CTLA-4 bispecific antibodies, which achieve intratumoural dual blockade; ③ antibody–drug conjugates, which are internalised through their target antigen and release a cytotoxic payload with bystander killing of neighbouring cells; ④ therapeutic HPV vaccines, which prime E6/E7-specific CD8^+^ T cells; ⑤ adoptive cell therapy, in which HPV-reactive tumour-infiltrating lymphocytes or E7-directed T-cell receptor–engineered T cells recognise E6/E7 peptides presented on MHC class I; and ⑥ antiangiogenic plus radio-immunotherapy combinations, which normalise aberrant vasculature and exploit radiation-induced immunogenic cell death. A single viral dependency thus creates six distinct therapeutic entry points. ADC, antibody–drug conjugate; APC, antigen-presenting cell; β2M, beta-2 microglobulin; CD8, cluster of differentiation 8; CIN, cervical intraepithelial neoplasia; CTLA-4, cytotoxic T-lymphocyte-associated protein 4; DNA, deoxyribonucleic acid; E5/E6/E7, HPV early proteins 5/6/7; HER2, human epidermal growth factor receptor 2; HLA-I, human leucocyte antigen class I; HPV, human papillomavirus; HSIL, high-grade squamous intraepithelial lesion; ICI, immune checkpoint inhibitor; IFN-γ, interferon gamma; JAK, Janus kinase; LAG-3, lymphocyte-activation gene 3; MDSC, myeloid-derived suppressor cell; MDR1, multidrug resistance protein 1; MHC-I, major histocompatibility complex class I; MMAE, monomethyl auristatin E; PD-1, programmed cell death protein 1; PD-L1, programmed death-ligand 1; P-gp, P-glycoprotein; pRb, retinoblastoma protein; R/M, recurrent/metastatic; RT, radiotherapy; TCR-T, T-cell receptor-engineered T-cell therapy; TF, tissue factor; TGF-β, transforming growth factor beta; TIGIT, T-cell immunoreceptor with immunoglobulin and ITIM domains; TIL, tumour-infiltrating lymphocyte; TIM-3, T-cell immunoglobulin and mucin-domain containing-3; TME, tumour microenvironment; Treg, regulatory T cell; Trop-2, trophoblast cell surface antigen 2; VEGF, vascular endothelial growth factor.

**Figure 2:**
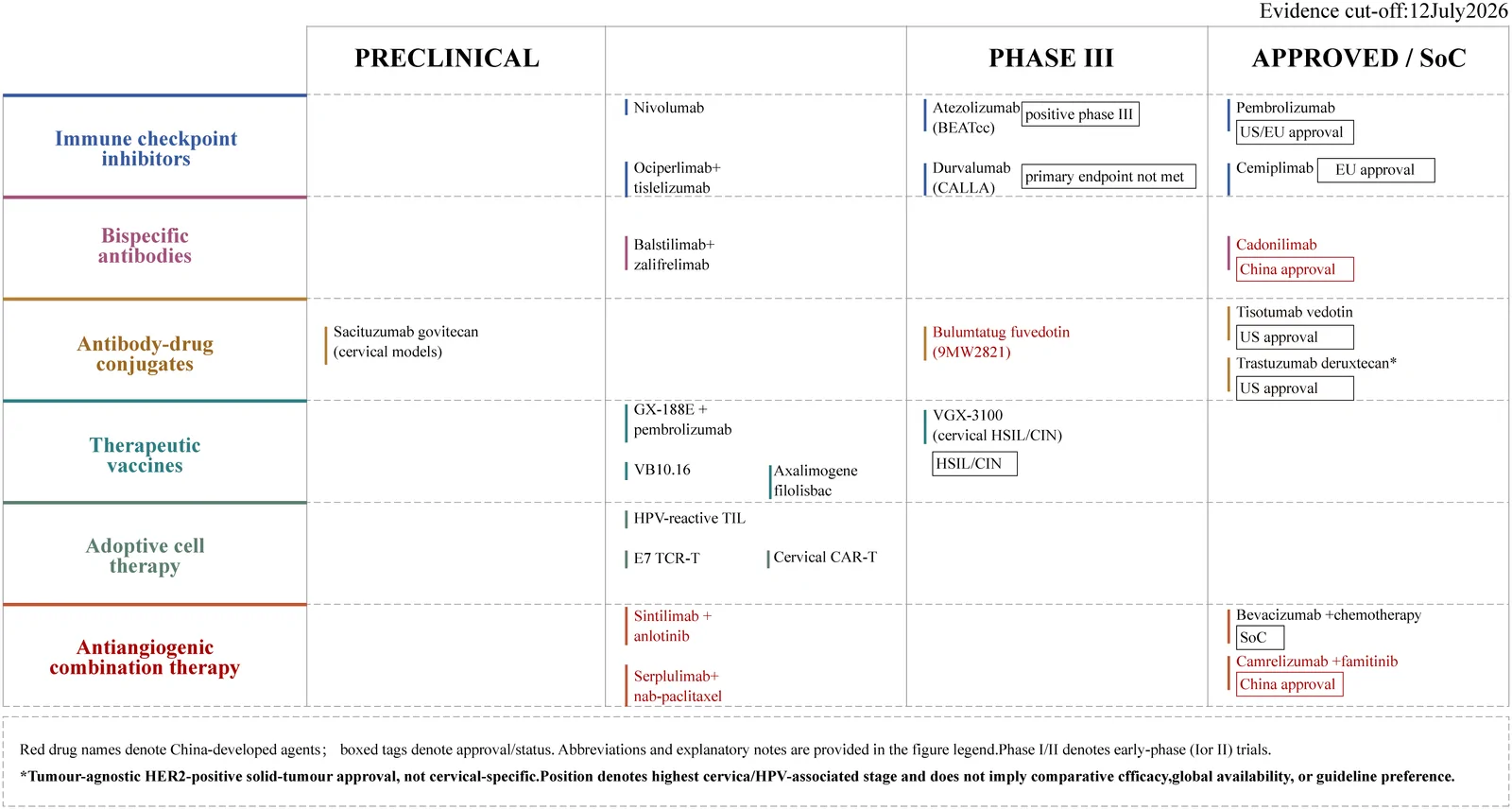
Translational maturity gradient of emerging anti-tumour agents across mechanism classes. Agents are positioned by their most advanced stage of clinical development—preclinical, phase 1/2, phase 3, or approved/standard-of-care—within mechanism-defined lanes (immune checkpoint inhibitors, bispecific antibodies, antibody–drug conjugates, therapeutic vaccines, adoptive cell therapy, and antiangiogenic/radio-immunotherapy combinations). arrows denote assets actively advancing towards a more mature stage. China-developed agents (including cadonilimab, 9MW2821, camrelizumab plus famitinib, sintilimab plus anlotinib, serplulimab and ociperlimab) are outlined in red. The distribution illustrates a mature checkpoint-inhibitor and antibody–drug-conjugate frontier at the approved end, an expanding middle of HER2-, Nectin-4- and combination-based strategies, and earlier-stage vaccine and cell-therapy assets, with China-developed evidence clustering in the bispecific, conjugate and combination lanes. CAR-T, chimaeric antigen receptor T-cell therapy; CIN, cervical intraepithelial neoplasia; E7, human papillomavirus early protein 7; EU, European Union; HER2, human epidermal growth factor receptor 2; HPV, human papillomavirus; HSIL, high-grade squamous intraepithelial lesion; nab-paclitaxel, nanoparticle albumin-bound paclitaxel; SoC, standard of care; TCR-T, T-cell receptor-engineered T-cell therapy; TIL, tumour-infiltrating lymphocyte; US, United States. Trial names: BEATcc, ENGOT-cx10/GOG-3029 trial; CALLA, NCT03830866 trial.

This review addresses that gap. We organise emerging anti-tumour agents by mechanism of action (**Figure 1**) and array them along a translational maturity gradient (**Figure 2**)—from approved regimens through phase 1/2 candidates to preclinical assets—whilst appraising efficacy and safety in parallel. In doing so, we integrate China-developed drugs into the global landscape and consolidate cross-class safety and biomarker considerations into a unified, clinically oriented framework intended to guide rational agent selection and, ultimately, the question of when in the treatment course each agent is best deployed.

## 2 Immune checkpoint inhibitors

Over the past decade, checkpoint blockade has advanced from a salvage option in platinum-refractory disease to an integral component of first-line and definitive-intent regimens, one of the largest shifts in the systemic management of the disease. The rationale is grounded in cervical tumour biology: high-risk HPV sustains constitutive expression of the viral oncoproteins E6/E7, frequent PD-L1 upregulation, and an inflamed but functionally exhausted T-cell infiltrate. Together, these features render the disease unusually susceptible to PD-1/PD-L1 inhibition. Successive randomised trials have converted this susceptibility into survival gains across the recurrent/metastatic (R/M) and locally advanced settings. At the same time, they have exposed the biomarker and resistance questions that now define the field.

Anti–PD-1 combined with platinum chemotherapy, with or without bevacizumab, has raised first-line R/M survival to levels not previously achieved. In KEYNOTE-826, adding pembrolizumab to chemotherapy ± bevacizumab produced concurrent overall survival (OS) and progression-free survival (PFS) benefits, with the benefit greatest in tumours with a high PD-L1 combined positive score (CPS) (11); the final analysis confirmed a durable OS advantage (median 28.6 vs 16.5 months in the CPS≥1 population; hazard ratio [HR] 0.60) (15), and prespecified subgroup analyses showed consistency across CPS strata and irrespective of bevacizumab use (16, 17). BEATcc extended this principle to a PD-L1–agnostic backbone, demonstrating that atezolizumab added to bevacizumab-containing chemotherapy improved both PFS and OS (median OS 32.1 vs 22.8 months; HR 0.68) (18). These trials established chemo-immunotherapy as the first-line standard (**Table 2**) and showed antiangiogenic co-administration to be a reproducible amplifier of its efficacy.

**Table 2:** Efficacy of approved and pivotal phase 3 agents in cervical cancer.

Agent (class)	Pivotal trial (phase)	Setting/population	Key efficacy (verified)	Regulatory/SoC status	Ref.
Pembrolizumab + chemotherapy ± bevacizumab (anti–PD-1)	KEYNOTE-826 (phase 3)	First-line R/M	Final mOS 28.6 vs 16.5 mo, HR 0.60 (CPS≥1); 26.4 vs 16.8 mo, HR 0.63 (ITT); 29.6 vs 17.4 mo, HR 0.58 (CPS≥10); mPFS 10.4 vs 8.2 mo, HR 0.62 (CPS≥1)	Standard of care	(11, 15–17)
Atezolizumab + bevacizumab–chemotherapy (anti–PD-L1)	BEATcc (phase 3)	First-line R/M (PD-L1-agnostic)	mPFS 13.7 vs 10.4 mo, HR 0.62; mOS 32.1 vs 22.8 mo, HR 0.68	Approved	(18)
Pembrolizumab + CCRT (anti–PD-1)	KEYNOTE-A18 (phase 3)	High-risk locally advanced (curative intent)	24-mo PFS 68% vs 57%, HR 0.70; 36-mo OS 82.6% vs 74.8%, HR 0.67 (mOS not reached)	Standard of care	(22, 23)
Cemiplimab (anti–PD-1)	EMPOWER-Cervical 1 (phase 3)	Second-line R/M after platinum	mOS 12.0 vs 8.5 mo, HR 0.69; mPFS HR 0.75; ORR 16.4% vs 6.3%; benefit PD-L1-agnostic	Approved	(19)
Cadonilimab + chemotherapy ± bevacizumab (PD-1/CTLA-4 bispecific, CN)	COMPASSION-16 (phase 3)	First-line R/M	mPFS 12.7 vs 8.1 mo, HR 0.62; mOS NR (27.0–NE) vs 22.8 mo, HR 0.64; benefit including PD-L1-negative	Approved (China)	(14, 35)
Tisotumab vedotin (TF-directed ADC, MMAE)	innovaTV 301 (phase 3)	Previously treated R/M	mOS 11.5 vs 9.5 mo, HR 0.70 (≈30% lower risk of death); mPFS 4.2 vs 2.9 mo, HR 0.67; ORR 17.8% vs 5.2% (accelerated approval on innovaTV 204 ORR 24% (39))	Approved	(12)
Bevacizumab + platinum–paclitaxel (anti-VEGF)	GOG-240 (phase 3)	Advanced/R/M	mOS 17.0 vs 13.3 mo, HR 0.71 (primary (8)); final 16.8 vs 13.3 mo, HR 0.77 (9); first non-cytotoxic survival gain	Standard of care	(8, 9)

ADC, antibody–drug conjugate; TF, tissue factor; MMAE, monomethyl auristatin E; VEGF, vascular endothelial growth factor. Efficacy values are verified against the primary trial publications (see Section 2–4, 7).

In the pretreated setting, single-agent anti–PD-1 delivers a survival benefit that does not depend on PD-L1 expression. EMPOWER-Cervical 1 showed an OS advantage for cemiplimab over investigator-choice chemotherapy (median 12.0 vs 8.5 months; HR 0.69) across the enrolled population, including PD-L1–unselected patients (19), whereas KEYNOTE-158 and CheckMate 358 had earlier defined the objective response rate and tolerability of pembrolizumab and nivolumab monotherapy in pretreated R/M disease (20, 21). These data anchor checkpoint monotherapy as the reference later-line option and provide the comparator against which combination and next-generation strategies are now measured.

Extending checkpoint blockade to definitive chemoradiotherapy (CCRT) has produced both a practice-changing success and an instructive failure. KEYNOTE-A18 showed that adding pembrolizumab to CCRT in high-risk locally advanced disease improved PFS and subsequently OS (36-month OS 82.6% vs 74.8%; HR 0.67), establishing a new standard for this curative-intent population (22, 23). CALLA, testing durvalumab with CCRT, was negative for PFS despite a superficially comparable design (24). The divergence reflects trial design more than the PD-L1 versus PD-1 axis: differences in patient risk enrichment, radiotherapy quality assurance, and the timing of immunotherapy relative to radiation plausibly account for the discordance. The window for radio-immunotherapy synergy thus appears narrow and design-sensitive.

PD-L1 CPS remains the principal enrichment tool, yet its limitations motivate a broader biomarker portfolio. Interobserver disagreement in CPS scoring of gynaecological specimens clusters near the positivity threshold, complicating threshold-based selection (25), and although PD-L1 expression carries prognostic weight in cervical squamous carcinoma (26), it predicts benefit only imperfectly. Tumour-intrinsic features—mismatch-repair deficiency/microsatellite instability—identify a smaller subset with durable, sometimes tissue-agnostic responses (27). HPV positivity, near-universal in cervical cancer, offers little discriminatory value, so contemporary selection increasingly integrates CPS with these orthogonal markers rather than relying on any single assay.

### 2.1 Resistance mechanisms and countermeasures

Because the anti-tumour effect of checkpoint blockade ultimately depends on intact interferon (IFN)-γ signalling and antigen presentation, the principal routes of resistance are those that dismantle this same axis. Loss-of-function mutations in JAK1/JAK2 are paradigmatic: by leaving tumour cells unable to respond to IFN-γ, they abolish reactive PD-L1 expression and antigen-processing machinery, and have been shown to drive both primary (28) and acquired (29) resistance, with truncating mutations in β2-microglobulin collapsing surface MHC-I through a parallel route. This dependence is not confined to PD-1 blockade, since copy-number loss of IFN-γ pathway genes (IFNGR1, IRF1, JAK2) similarly abrogates the response to CTLA-4 inhibition (30); IFN-γ sensing is therefore a shared point of failure across checkpoint classes. In cervical cancer this genetic vulnerability is layered onto a virus-driven one: the HPV-16 E5 oncoprotein sequesters HLA class I within the Golgi and diminishes its surface display, weakening CD8 recognition even where the antigen-presentation genes remain intact (31). And where the tumour-cell machinery is preserved, the microenvironment can still re-impose exhaustion, as regulatory T cells (Tregs), myeloid-derived suppressor cells (MDSCs) and TGF-β act together with the adaptive upregulation of alternative immune checkpoints, most notably TIM-3, to blunt the response despite continued PD-1 engagement (32). This network, rather than the checkpoint target, sets the ceiling of chemo-immunotherapy. Dense Treg and MDSC infiltration with active TGF-β signalling largely excludes effector T cells from the tumour. The first-line gains recorded in KEYNOTE-826 and BEATcc are therefore unlikely to be realised in full. TIM-3 upregulation on tumour-infiltrating lymphocytes then restores exhaustion under continued treatment, and some early responses become progression (32). Each of these mechanisms points towards a matched countermeasure. Compensatory checkpoint upregulation is the most directly druggable, and is under clinical test in cervical cancer through the China-developed anti-TIGIT (T-cell immunoreceptor with immunoglobulin and ITIM domains) antibody ociperlimab combined with tislelizumab in the randomised AdvanTIG-202 trial (NCT04693234), whilst LAG-3 blockade lends proof of concept, having reached regulatory approval alongside nivolumab in melanoma (RELATIVITY-047) (33) before its ongoing extension into solid tumours. Where the tumour cell itself is the barrier, the more tractable strategy is to bypass rather than restore it—switching to antibody–drug conjugates on checkpoint failure, or bridging single-agent PD-1 resistance with bispecific co-inhibition—an approach that carries these mechanistic insights into the combination strategies detailed below.

The trajectory of checkpoint inhibition in cervical cancer has moved from proof of activity to platform validation across the disease continuum, yet its ceiling is now defined by this resistance biology. The unresolved questions are translational rather than confirmatory: how to prospectively identify the CPS-low, IFN-γ–disrupted tumours destined to fail PD-1 blockade; how to pharmacologically reverse HPV-driven antigen-presentation defects; and how to sequence or combine checkpoint agents with antiangiogenics, ADCs, bispecifics and radiation for maximal and durable benefit. Progress will depend on biomarker frameworks that extend beyond PD-L1 CPS and on trial designs that treat resistance as a predictable, mechanistically defined endpoint rather than an incidental observation.

## 3 Bispecific antibodies

Bispecific antibodies that simultaneously engage PD-1 and CTLA-4 are designed to consolidate two complementary checkpoint pathways within a single molecule, exploiting the higher CTLA-4 density on tumour-infiltrating lymphocytes to concentrate dual blockade within the tumour whilst limiting the systemic toxicity that has constrained conventional anti–CTLA-4 antibodies. Cadonilimab (AK104), a China-developed tetravalent PD-1/CTLA-4 bispecific, is the most clinically advanced example, and its phase 2 COMPASSION-13 experience in recurrent/metastatic (R/M) cervical cancer supplied the activity and tolerability signal that justified first-line testing (34).

Building on that signal, cadonilimab combined with platinum chemotherapy became the first PD-1/CTLA-4 bispecific to demonstrate a first-line overall survival (OS) benefit in R/M cervical cancer. In the phase 3 COMPASSION-16 trial, adding cadonilimab to chemotherapy ± bevacizumab improved both progression-free survival and OS (median PFS 12.7 vs 8.1 months, HR 0.62; median OS not reached vs 22.8 months, HR 0.64), and—unusually for a population routinely stratified by PD-L1—the benefit extended to PD-L1–negative tumours (14), a pattern reinforced by consistency across prespecified subgroups (35). The agent thus emerged as a first-line option less dependent on biomarker enrichment than single-agent PD-1 approaches.

The strength of this OS conclusion nonetheless warrants measured interpretation. Independent commentary, whilst affirming the positive result, has cautioned that whether adding dual PD-1/CTLA-4 blockade to chemotherapy confers benefit beyond established chemo-immunotherapy is not yet settled: cross-trial comparison with those regimens is confounded, and the apparent efficacy in PD-L1–negative tumours rests on a small subgroup (36). This argues for longer, updated and ideally comparative analyses before the incremental value of dual over single checkpoint blockade can be regarded as established.

Beyond cadonilimab, earlier-phase dual-checkpoint combinations corroborate the platform, as balstilimab paired with the anti–CTLA-4 antibody zalifrelimab produced measurable second-line activity (37), including occasional abscopal responses when combined with radiation (38). The resistance behaviour of these agents is largely inherited from single-agent checkpoint blockade, since tumours with disrupted IFN-γ sensing remain refractory however many surface checkpoints are engaged (28); what distinguishes the bispecific design is a specific rationale against adaptive resistance, in that the added CTLA-4 arm can partly counter the compensatory checkpoint upregulation that erodes durable responses to PD-1 monotherapy (32). The advantage is only partial, because dual blockade leaves the cellular and cytokine arms of immunosuppression intact. Tregs and MDSCs continue to exclude effector T cells, and TGF-β signalling disarms those that remain. Checkpoints other than CTLA-4, including TIM-3, are also still upregulated under sustained co-inhibition (32). This may explain both the early progressions recorded in COMPASSION-16 and the difficulty of interpreting the PD-L1–negative signal (14, 36). Dual targeting therefore neutralises one resistance axis whilst remaining exposed to the tumour-intrinsic routes described in Section 2.

PD-1/CTLA-4 bispecifics are accordingly best positioned as a biomarker-agnostic option for PD-L1–low disease and a rational partner in resistance-directed regimens, rather than as a universal replacement for single-agent checkpoint blockade. Whether their dual mechanism yields a reproducible survival advantage over chemo-immunotherapy, and in whom, remains the central unresolved question—one that mature comparative data, and biomarkers able to identify CTLA-4–driven rather than antigen-presentation–driven resistance, will be needed to answer.

## 4 Antibody–drug conjugates

Antibody–drug conjugates (ADCs) have moved from concept to validated platform in cervical cancer, coupling the selectivity of a targeting antibody to the potency of a cytotoxic payload through a chemically defined linker. Their appeal in this disease rests on two features. The first is the surface expression of exploitable antigens (tissue factor [TF], HER2, Nectin-4 and Trop-2) on a large fraction of cervical tumours. The second is the bystander effect: a membrane-permeable payload released within antigen-positive cells also kills neighbouring antigen-low cells, which partly offsets the antigenic heterogeneity that characterises the disease. The regulatory approval of a TF-directed ADC has anchored the class, and a widening pipeline is now testing whether alternative targets and payloads can extend its reach.

Tisotumab vedotin, a TF-directed antibody linked through a protease-cleavable valine–citrulline linker to the microtubule inhibitor monomethyl auristatin E (MMAE), is the validating agent. TF is expressed on a large proportion of cervical carcinomas, providing a broad therapeutic target (13), and the drug’s activity reflects a multimodal mechanism—direct cytotoxicity, MMAE-driven bystander killing, immunogenic cell death and Fc-mediated effector functions. After the single-arm innovaTV 204 study established a reproducible objective response rate (24%) and supported accelerated approval (39), the randomised phase 3 innovaTV 301 trial confirmed an overall survival (OS) advantage over chemotherapy (median 11.5 vs 9.5 months; HR 0.70) in previously treated R/M disease (12), and innovaTV 205 has extended evaluation to first-line combinations with carboplatin, pembrolizumab or bevacizumab (40).

Beyond TF, HER2 offers the most clinically mature alternative target. Trastuzumab deruxtecan (T-DXd), which pairs an anti-HER2 antibody with a topoisomerase-I inhibitor payload, produced meaningful responses in HER2-expressing cervical cancer within the tissue-agnostic DESTINY-PanTumor02 basket (41), with the gynaecological-cohort analysis reporting a 50% objective response rate in the cervical cohort and an IHC-stratified *post hoc* analysis refining which HER2 expression levels predict benefit (42, 43). These data are clinically grounded, since HER2 amplification occurs in a defined subset of cervical adenocarcinomas (44) and HER2 overexpression is also documented in squamous histology (45), so that HER2 represents a testable, prevalence-supported target.

Two further targets illustrate the platform’s expansion and the contribution of China-developed agents. Nectin-4, validated as an ADC target in urothelial cancer, is pursued in cervical cancer by the China-developed conjugate 9MW2821, for which preclinical characterisation and structural definition of Nectin-4 recognition support early-phase clinical testing (46, 47). Trop-2 is addressed preclinically by sacituzumab govitecan, whose activity tracks with high Trop-2 expression (48). The rational selection of these targets is increasingly informed by expression mapping: co-expression profiling of TF, Trop-2 and Nectin-4 across primary and metastatic cervical specimens provides the prevalence data needed to match target to patient (13).

The class carries characteristic, payload- and target-linked toxicities that shape its clinical use. In the pivotal tisotumab vedotin trials, ocular adverse events, chiefly conjunctivitis and keratitis, were amongst the most common toxicities and are mitigated by a mandated ocular-care protocol and dose modification (39), whilst bleeding events and peripheral neuropathy, consistent respectively with the physiological role of tissue factor in coagulation and with the antimitotic payload, were also characteristic (12). These profiles are largely manageable but distinct from the immune-related toxicities of checkpoint agents, so ADC selection must weigh organ-specific risk alongside target prevalence.

### 4.1 Resistance mechanisms and countermeasures

Because ADC cytotoxicity depends on an intact delivery cascade—antigen binding, internalisation, lysosomal processing, payload release and intracellular payload retention—resistance can arise at any step along that chain. The most intuitive route is antigen-level failure, as downregulation or heterogeneous loss of the target antigen reduces drug delivery; in cervical cancer, however, the bystander effect of membrane-permeable MMAE partly compensates, so that preclinical TF-ADC activity is retained even in tumours expressing the target in only a minority of cells (49), blunting the impact of antigenic heterogeneity. When the tumour cell instead defeats the payload after delivery, drug efflux becomes dominant: MMAE is a substrate of the P-glycoprotein/MDR1 transporter, and its upregulation on plasma and lysosomal membranes actively exports the payload to confer resistance, an effect reversible *in vitro* by transporter inhibition or lysosomal disruption (50). A related route lies in linker processing and intracellular trafficking, where defective lysosomal cleavage limits payload liberation; the pattern is informative because tumour cells chronically exposed to a non-cleavable maytansinoid ADC become cross-resistant to similar conjugates yet retain sensitivity to cleavable auristatin-linked ADCs (51). That dissociation directly motivates the principal countermeasures—switching payload class or linker chemistry, changing the target antigen, and sequencing distinct ADCs rather than re-challenging with a cross-resistant one—whilst the bystander effect itself functions as a built-in design hedge against antigen heterogeneity. Resistance also arises outside the tumour cell, in the microenvironment. Part of the activity of MMAE-based conjugates depends on immunogenic cell death and Fc-mediated effector function, both of which need competent immune effectors. A TGF-β–conditioned, desmoplastic stroma limits antibody penetration, whilst Tregs, MDSCs and TIM-3 upregulation suppress that immune contribution (32). This constrains what checkpoint blockade can add when ADCs and immunotherapy are combined.

The ADC platform is thus established in cervical cancer, but its durability now depends on target selection and resistance biology, no longer on proof of principle. Three problems now govern its trajectory: quantifying target expression and heterogeneity prospectively to guide agent choice; countering payload efflux and trafficking defects pharmacologically; and sequencing ADCs of differing payloads, and combining them with checkpoint blockade, so that the platform’s several targets act as a coordinated resistance-directed strategy instead of interchangeable single agents. Answering these will determine whether the expanding pipeline of TF, HER2, Nectin-4 and Trop-2 conjugates delivers additive benefit or merely additional alternatives.

## 5 Therapeutic HPV vaccines

Unlike the prophylactic vaccines that target the capsid protein L1 to prevent infection, therapeutic HPV vaccines are designed to treat established disease by directing cellular immunity against the E6 and E7 oncoproteins. These antigens are near-ideal targets: they are constitutively expressed, functionally required to maintain the malignant phenotype, and genuinely foreign, so that the immune tolerance limiting responses to self-antigens does not apply. In practice, this rationale translates most readily into clinical benefit in the pre-invasive setting, where antigen burden is low and the immunosuppressive tumour microenvironment is not yet fully established, and only modestly in invasive R/M disease. This efficacy gradient has reframed these agents from stand-alone therapeutics into combination partners.

The clearest proof of concept comes from DNA-based platforms in high-grade cervical intraepithelial neoplasia (CIN). VGX-3100, a plasmid encoding HPV-16 and HPV-18 E6/E7 delivered by electroporation, was the first therapeutic HPV vaccine to demonstrate histological regression of CIN2/3 in a randomised, placebo-controlled phase 2b trial, establishing that a vaccine alone can reverse premalignant disease (52). Subsequent constructs have pursued greater potency through antigen-presenting-cell targeting, as with VB10.16, which produced lesion regression and viral clearance in HPV-16-positive high-grade CIN (53). Peptide and vector-based approaches have followed the same premalignant-first trajectory. A synthetic long-peptide vaccine covering the full E6/E7 sequence achieved durable complete responses in vulvar intraepithelial neoplasia, the landmark demonstration that a defined peptide immunogen can clear HPV-driven lesions (54). Alphavirus-replicon and Listeria-based vectors have since been engineered to amplify E6/E7-specific T-cell priming (55, 56).

Extension to invasive disease has exposed the ceiling of vaccination as monotherapy. When the same synthetic long peptides were given to patients with end-stage cervical cancer, they were immunogenic and well tolerated but induced no meaningful tumour regression; the block lay in the established immunosuppressive microenvironment, not in failed priming (57). That block is cellular and cytokine-mediated rather than antigenic in origin. Tregs and MDSCs accumulate within HPV-driven lesions and, together with TGF-β, suppress vaccine-primed effectors at the tumour site. E6/E7-specific T cells that expand in blood also upregulate TIM-3 and related checkpoints once they enter the lesion (32, 57). Immunogenicity is measurable, but it is not converted into regression. Listeria-vectored axalimogene filolisbac met a pre-specified survival benchmark in platinum-refractory disease within the GOG-0265 study, but its single-agent activity remained limited (58). These results have redirected development towards rational combination, and the most influential signal is the pairing of the DNA vaccine GX-188E with pembrolizumab, in which vaccine-primed E6/E7-specific T cells and checkpoint blockade to sustain their function produced responses in advanced disease exceeding those expected from either component alone (59).

Therapeutic HPV vaccination is therefore best understood as a mechanistically validated platform whose clinical role is context-dependent: near-curative potential in high-grade CIN, where it offers a non-surgical alternative, and an adjunctive, immune-priming role in invasive cancer, where its future lies in combination with checkpoint inhibitors and, plausibly, with adoptive cell therapy. The toxicity profile is consistently favourable, dominated by transient injection-site and flu-like reactions, which makes these agents attractive combination backbones. Two questions remain central: which platform and delivery method generate the most functional intratumoural T cells, and how best to schedule vaccination relative to checkpoint blockade so that antigen priming and de-repression of the resulting response are optimally aligned.

## 6 Adoptive cell therapy

Adoptive cell therapy exploits the same viral-antigen advantage as therapeutic vaccination but delivers effector T cells directly, bypassing the need to prime an endogenous response in an immunosuppressed host. Across platforms, clinical benefit tracks with the reactivity of the infused product against HPV E6/E7. Cervical cancer, an obligately antigen-expressing malignancy, is therefore a rational target for cellular therapies, whose main constraints are manufacturing complexity and accessibility rather than antigen availability.

Tumour-infiltrating lymphocyte (TIL) therapy provided the first proof that a cellular product can eradicate an epithelial solid tumour. In a landmark study, a single infusion of HPV-reactive TILs produced durable complete regressions in metastatic cervical cancer, and the depth of response correlated with the frequency of HPV-specific T cells within the product (60). A subsequent phase 2 experience confirmed objective responses and reinforced the link between HPV reactivity and efficacy, whilst also exposing the heterogeneity of the polyclonal TIL approach (61). Whether centralised, commercial-scale manufacturing can reproduce these results in cervical cancer beyond specialised academic centres is an open question that will determine the approach’s real-world reach.

Genetically engineered T cells offer a more defined alternative by conferring a single, known specificity. A T-cell receptor (TCR)-transduced product directed against the HPV-16 E7 epitope achieved objective responses in heavily pre-treated patients, demonstrating that a uniform, on-target-antigen-specific population can mediate regression and providing a more reproducible manufacturing template than TIL expansion (62). Chimeric antigen receptor (CAR)-T approaches, dependent on surface rather than intracellular targets, remain largely preclinical in this disease, since the defining HPV oncoproteins are processed intracellularly and presented on MHC, favouring TCR- over CAR-based recognition.

The barriers to durable benefit are shared across platforms and define the resistance and relapse biology of the class. Antigen escape through loss of the targeted E7 epitope or downregulation of MHC-I presentation removes the very signal these therapies depend on; infused T cells are further limited by exhaustion and inadequate *in vivo* persistence, and by a suppressive microenvironment that restricts intratumoural infiltration—the same immunosuppressive context that blunts vaccination in invasive disease. Within that context, Tregs, MDSCs and TGF-β disable the transferred product, and TIM-3 upregulation reinstates exhaustion in the cells that do reach the tumour (32).

Adoptive cell therapy thus offers the highest-magnitude single-agent responses documented for HPV-driven cancer but at the cost of individualised manufacturing, lymphodepleting conditioning and specialised infrastructure that constrain global accessibility. Its trajectory mirrors that of the vaccines, and its priorities follow directly: sustaining effector persistence, countering antigen and MHC-I escape, and combining cellular products with checkpoint blockade or vaccination so that on-target specificity is matched by durable function within the tumour.

## 7 Other targeted agents and rational combinations

Beyond the antibody- and cell-based platforms discussed above, progress in cervical cancer increasingly depends on rational combinations built on two mechanistic backbones—tumour angiogenesis and radiation-induced immunogenicity—that convert individually modest agents into synergistic regimens.

Antiangiogenic therapy remains the foundational backbone. As introduced above, the addition of bevacizumab to platinum chemotherapy in GOG-240 was the first strategy to extend overall survival in advanced disease and established vascular endothelial growth factor (VEGF) blockade as a standard partner (8), albeit at the cost of characteristic toxicities—hypertension, thromboembolism and gastrointestinal–genitourinary fistulae—that require careful patient selection (9). Its mechanistic value extends beyond growth inhibition: by normalising aberrant tumour vasculature and relieving hypoxia-driven immunosuppression, VEGF blockade creates a microenvironment more permissive to immune attack, providing the rationale for the antiangiogenic–checkpoint combinations that now anchor first-line care (Section 2). The remodelling is incomplete, however, because Tregs, MDSCs and TGF-β signalling persist under VEGF blockade and continue to limit what antiangiogenic–immunotherapy combinations achieve.

This principle has been most actively extended by China-developed regimens pairing checkpoint inhibitors with oral multikinase inhibitors. Camrelizumab combined with the antiangiogenic tyrosine kinase inhibitor famitinib produced encouraging activity in recurrent or metastatic squamous disease (63), and sintilimab plus anlotinib generated durable responses that were sustained on three-year follow-up, indicating that chemotherapy-free doublets can achieve meaningful and lasting disease control (64, 65). Parallel efforts have paired domestic checkpoint antibodies with chemotherapy, as with serplulimab or sintilimab combined with nab-paclitaxel (66, 67), broadening the combination landscape whilst reflecting the increasingly central role of China-developed agents in this disease.

Radiation provides the second synergistic axis. Beyond its cytoreductive role, radiotherapy—particularly when fractionated—can prime systemic, abscopal anti-tumour immunity that is unmasked by concurrent checkpoint blockade, which provides the mechanistic basis for radio-immunotherapy (68). This approach is validated in the locally advanced setting (Section 2) and is now being explored in the neoadjuvant window, where tislelizumab added to chemotherapy has shown activity before definitive treatment (69).

These combinations link single-platform activity into coordinated regimens. What remains unsettled is which backbone to prioritise for a given clinical context, how to sequence combinations against emerging resistance, and how to identify the biomarkers that will spare patients the additive toxicity of regimens from which they are unlikely to benefit.

## 8 Cross-cutting considerations: safety landscape and biomarkers

A practically important feature of the modern armamentarium is that its toxicities are largely mechanism-specific and non-overlapping (**Table 3**), which makes the safety profile itself a determinant of agent selection. Checkpoint inhibitors produce immune-related adverse events that can affect virtually any organ and require immunosuppression-based management, as characterised in the randomised cemiplimab trial in recurrent cervical cancer (19); bispecific PD-1/CTLA-4 blockade intensifies this same immune toxicity through dual co-inhibition. In contrast, antibody–drug conjugates carry payload- and target-linked effects—ocular surface injury, bleeding and peripheral neuropathy (Section 4)—whilst adoptive cell therapy adds the risks of lymphodepleting conditioning. Because these profiles are largely non-overlapping, sequential and combination regimens can often be assembled without compounding a single organ toxicity, provided the dominant risk of each class is anticipated.

**Table 3:** Cross-class safety, resistance mechanisms and predictive biomarkers.

Agent class	Characteristic toxicities and management	Principal resistance mechanisms	Predictive/selection biomarkers	Ref.
ICI	Immune-related adverse events affecting virtually any organ; immunosuppression-based management (19)	Failure of IFN-γ sensing: JAK1/2 loss-of-function (primary (28)/acquired (29)), β2-microglobulin truncation with MHC-I loss, copy-number loss of IFN-γ pathway genes (30); HPV E5–mediated Golgi retention of HLA class I (31); suppressive microenvironment with Treg/MDSC infiltration, TGF-β signalling and adaptive TIM-3/LAG-3/TIGIT upregulation (32)	PD-L1 CPS (limited interpretive concordance and prognostic value (25, 26)); MSI-high/dMMR small subset (27)	(19, 25–32)
Bispecific (PD-1/CTLA-4)	Immune toxicity intensified by dual co-inhibition	Shares IFN-γ–dependent resistance with ICIs (28); the CTLA-4 arm may partly offset adaptive checkpoint upregulation (32); Treg/MDSC and TGF-β–mediated suppression unaffected by dual blockade	Less dependent on PD-L1 than single-agent PD-1 blockade (14)	(14, 28, 32)
ADC	Ocular surface injury (conjunctivitis/keratitis; mandatory ophthalmic prophylaxis) (39); bleeding and peripheral neuropathy (12)	Target-antigen downregulation/heterogeneity [partly offset by bystander effect (49)]; MMAE efflux via MDR1/P-gp (50); linker-cleavage/trafficking defects causing cross-resistance (51); TGF-β–driven desmoplasia limiting antibody penetration, with Treg/MDSC and TIM-3–mediated suppression of the immune component of ADC activity (32)	Target-antigen expression (TF/HER2/Nectin-4/Trop-2; thresholds not yet standardised) (13, 42, 43)	(12, 13, 32, 39, 42, 43, 49–51)
Therapeutic vaccine	Transient injection-site and influenza-like reactions (favourable profile)	Established immunosuppressive microenvironment of invasive disease limits efficacy (not a failure of priming) (57); intratumoural Treg/MDSC and TGF-β suppression, with TIM-3 upregulation on vaccine-primed T cells (32)	HPV genotype/E6–E7 status; lesion stage (CIN vs invasive)	(32, 57)
Adoptive cell therapy	Risks of lymphodepleting conditioning	E7-epitope loss/MHC-I downregulation; T-cell exhaustion and insufficient persistence; suppressive microenvironment limiting infiltration (Treg/MDSC, TGF-β; TIM-3 upregulation on transferred cells) (32)	HPV reactivity of the infused product (60); MHC-I integrity	(32, 60)
Antiangiogenic/combinations	Hypertension, thromboembolism, gastrointestinal–genitourinary fistula (careful patient selection) (9)	Functions as a backbone; no independent resistance column (see individual combination sections)	Determined by the biomarkers of the partner agent	(9)

IFN-γ, interferon-γ; MHC-I, major histocompatibility complex class I; MSI, microsatellite instability; dMMR, mismatch-repair deficiency; MDR1/P-gp, multidrug resistance protein 1/P-glycoprotein; TF, tissue factor; CIN, cervical intraepithelial neoplasia. Resistance details are consolidated here to avoid repetition in the main text; mechanistic detail is cross-referenced to Sections 2, 4 and 6.

Biomarker development remains the rate-limiting step for precision use, hampered above all by the absence of any single assay that serves the whole armamentarium. For checkpoint blockade, PD-L1 CPS remains the principal predictive tool, with the reproducibility and prognostic limitations—and the small, tissue-agnostic MSI-H/dMMR subset—detailed in Section 2. Antigen-directed agents depend instead on target expression—tissue factor, HER2, Nectin-4, Trop-2—and on HPV antigen status, yet standardised, prospectively validated thresholds are lacking for any of these. The biomarker landscape is therefore fragmented across classes, and rational selection must currently pair an imperfect immunotherapy predictor with semi-quantitative target-expression assays for the antibody- and cell-based platforms.

**Figure 3:**
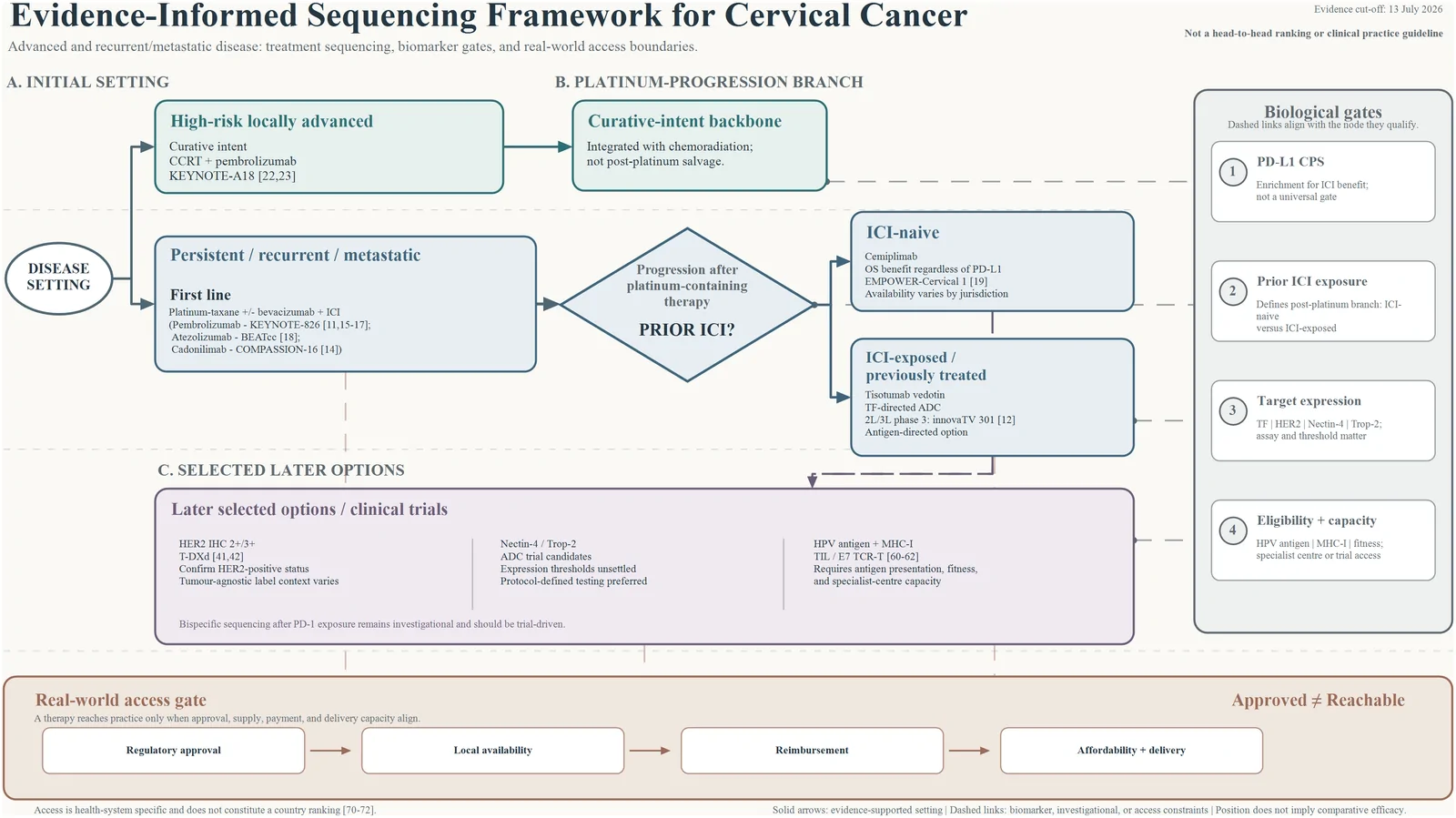
A mechanism- and biomarker-guided sequencing framework, bounded by real-world accessibility. Agents are mapped to clinical decision points along the treatment course: from locally advanced disease (concurrent chemoradiotherapy plus pembrolizumab), through first-line recurrent or metastatic disease (platinum-based chemotherapy with pembrolizumab, cadonilimab or atezolizumab, stratified where relevant by PD-L1 combined positive score), to the platinum-resistant setting. At this decisive branch point, prior checkpoint exposure and target-antigen expression direct the choice amongst checkpoint monotherapy (cemiplimab in checkpoint-naïve patients), antibody–drug conjugates (tisotumab vedotin in checkpoint-exposed patients, and alternative-target conjugates in later lines), bispecific co-inhibition and adoptive cell therapy. Diamond decision gates mark the biomarker and prior-therapy criteria that govern each choice. An overlaid accessibility–reimbursement gradient indicates how the line of therapy a patient can actually reach may be constrained by cost and insurance coverage rather than by tumour biology—an approved agent is not necessarily a reachable one—most acutely in the low- and middle-income settings that carry the greatest disease burden. 2L/3L, second-line/third-line; ADC, antibody–drug conjugate; CCRT, concurrent chemoradiotherapy; CPS, combined positive score; E7, human papillomavirus early protein 7; HER2, human epidermal growth factor receptor 2; HPV, human papillomavirus; ICI, immune checkpoint inhibitor; IHC, immunohistochemistry; MHC-I, major histocompatibility complex class I; Nectin-4, nectin cell adhesion molecule 4; OS, overall survival; PD-1, programmed cell death protein 1; PD-L1, programmed death-ligand 1; TCR-T, T-cell receptor-engineered T-cell therapy; T-DXd, trastuzumab deruxtecan; TF, tissue factor; TIL, tumour-infiltrating lymphocyte; Trop-2, trophoblast cell surface antigen 2.

These considerations converge on sequencing, nowhere more consequentially than in platinum-resistant or platinum-refractory recurrent/metastatic disease—the point at which cytotoxic options are largely exhausted and the agents reviewed here become clinically decisive. Because resistance mechanisms are class-specific (detailed by mechanism above), failure of one platform need not preclude another. For patients who progress after platinum without prior checkpoint exposure, second-line cemiplimab confers an overall survival benefit irrespective of PD-L1 status and represents a default option (19). As first-line pembrolizumab-based regimens become standard, however, a growing proportion of platinum-resistant patients are also checkpoint-exposed. For this population tisotumab vedotin is pivotal: its phase 3 survival advantage was demonstrated specifically in previously treated patients, including those refractory to both platinum and prior immunotherapy, through cytotoxicity that depends on antigen delivery rather than on immune function (12). Later lines can then draw on alternative-target antibody–drug conjugates, bispecific co-inhibition to bridge adaptive PD-1 resistance, and adoptive cell therapy. The platinum-resistant pathway is thus the clearest real-world expression of the resistance-directed sequence this review advocates (**Figure 3**), turning the mechanistic diversity of the pipeline into a coherent, biomarker- and prior-therapy-guided treatment algorithm. In practice this biologically rational sequence is bounded by a further, non-biological constraint: an agent that is approved is not necessarily reimbursed, and the line at which a patient can actually reach it may be dictated as much by cost and insurance coverage as by tumour biology or prior therapy (70).

## 9 Discussion, conclusion and future directions

Across the mechanistic spectrum surveyed here, cervical cancer has moved within a decade from a disease with a single antiangiogenic advance to one served by a diversified pipeline spanning checkpoint inhibitors, bispecific antibodies, antibody–drug conjugates, therapeutic vaccines and adoptive cell therapy. What unites these otherwise disparate platforms is the biology of HPV: viral oncoproteins, a consequently immunogenic microenvironment and a defined set of exploitable surface antigens together explain why immune- and antigen-directed strategies have succeeded where they have. The field has largely completed its phase of platform validation.

The task ahead is integration more than discovery. The maturity gradient makes this concrete: checkpoint blockade and the tissue-factor conjugate have reached the standard of care, HER2-, Nectin-4- and Trop-2-directed conjugates and combination immunotherapy occupy the emerging middle, and vaccines and engineered cell therapies remain earlier but mechanistically compelling. Converting this breadth into durable benefit will require three advances: prospectively validated, standardised biomarkers that extend beyond PD-L1 to antigen expression and antigen-presentation integrity; resistance-directed sequencing that treats the class-specific mechanisms detailed above as predictable phenomena; and rational combinations (checkpoint plus conjugate, vaccine plus checkpoint) designed to pre-empt escape instead of salvaging it.

Two themes will shape the coming years, and both expose the limits of an approval-driven view of progress. The first concerns where evidence is now generated: China-developed agents, amongst them cadonilimab, 9MW2821 and an expanding roster of domestic combinations, have become central to the global evidence base, diversifying the pipeline geographically as well as mechanistically. The second, and more fundamental, is the gap between guidelines and practice. Because current recommendations rest on phase 3 trials conducted in resource-rich settings, whilst access to newly approved agents varies enormously between health systems, the de facto standard of care, and therefore the line of therapy at which any novel agent is introduced, differs markedly across the world. That variation is driven less by clinical evidence than by economics: even medicines that oncologists judge essential are frequently unavailable or financially catastrophic for patients in low- and middle-income countries, precisely the settings that carry the greatest cervical cancer burden (70). The price and reimbursement status of an agent thus effectively determines the line at which, if at all, it can be used. This is why value frameworks that grade the magnitude of clinical benefit to inform resource-allocation and reimbursement decisions have become integral to rational agent selection (71). Regional development and access to that development intersect here: China’s national reimbursement negotiations, by listing anticancer agents at substantially reduced prices, have measurably improved the accessibility of high-cost therapy (72), one route by which regionally originated drugs can widen access where imported agents remain cost-prohibitive. This is decision-relevant, not merely a question of equity, because the benefit of these agents appears to be sequence-dependent: first-line checkpoint integration reshaped overall survival (11) to a degree that later-line monotherapy has not (20). This contrast is necessarily confounded, since line of therapy, patient selection and single-agent-versus-combination design differ simultaneously across these trials; it should therefore be read not as evidence that line of therapy independently determines survival, but as motivation for studies designed to test it directly. Whether introducing a given agent earlier, rather than after several prior lines, itself alters survival is therefore a question that approval status alone cannot answer, and one that regional variation in baseline care renders globally heterogeneous. The field consequently needs sequencing-aware, context-adapted evidence, together with trial designs that treat line of therapy and local standard of care as variables rather than constants, so that the ultimate measure of these advances, whether their benefit reaches patients where cervical cancer is most lethal (5, 73), is met in practice and not only in principle.
